# Supplementary material for: Premature cognitive decline in specific domains found in young veterans with mTBI coincide with elder normative scores and advanced-age subjects with early-stage Parkinson’s disease
Source: PLoS One. 2021 Nov 17;16(11):e0258851. doi: 10.1371/journal.pone.0258851 (PMC8598036; doi:10.1371/journal.pone.0258851)
Supplement: Attachment — Submitted filename: Response to Reviewers_Final.docx [file pone.0258851.s002.docx]

Firas H Kobeissy, PhD

Academic Editor

PLOS ONE

PONE-D-21-11322R1
Premature Cognitive Aging in Specific Domains in Young Veterans with mTBI
Coincide with Early-Stage Parkinson’s Disease [Nejtek et al.]

Dear Dr. Kobeissy,

Thank you for allowing my co-authors and me to revise and resubmit our manuscript to PLOS ONE. Here, we are submitting our rebuttal letter that responds to each point raised by the you as academic editor and the other reviewers. We have also included the marked-up copy of the manuscript and an unmarked version.

In terms of journal requirements, we have added 2 references which we included in the revised text and reference list.

Thank you for your kind consideration of publishing our manuscript should the revisions meet with your approval.

Sincerely,

Vicki

Additional Editor Comments:

1. There is definitely no study out there that indicates that 56% of Veterans develop PD within 4.6-6.8 years of mTBI. If this were the case, we would have a massive epidemic of PD right now. In fact, PD is a relatively rare disease with incidence <<1%, even after TBI. Perhaps the authors mis-understood an epidemiological study that may have identified 56% increased RISK of PD among Veterans with mTBI.

***Author’s Response***

***We sincerely appreciate and thank the Editor for pointing out our unintended omission of RISK in the first sentence in the Abstract. We surely did not intend to insinuate that there was this overwhelmingly high number of Parkinson’s disease affecting our veterans. We have revised the Importance section in the Abstract lines 30-34. After careful review of our paper, we found in the Introduction, the first sentence correctly stated the 56% increased risk in lines 56-57, and we found no other miscommunications on the ‘risk’ aspect in rest of the paper.***

2. The finding that in this cross-sectional study, Veterans with TBI perform worse than Veterans without TBI does not equal premature "cognitive aging" as the authors have not demonstrated any progressive cognitive decline. Perhaps the Veterans with TBI simply have a static injury that is resulting in worse cognitive performance. I would not equate this with cognitive aging, which is generally a term used to indicate a progressive decline in cognition associated with aging.

***Author’s Response***

***Many thanks to the Editor regarding their insight concerning the traditional use of the term ‘cognitive aging’. Although we did not run a longitudinal study and cannot predict measure progression, we used the TMT-A and B which has been investigated as a significantly sensitive predictor of progression to dementia within 4.3- to 6-years [DeRoy et al., 2020; Marchand et al, 2018, respectively] (p. 17, lines 419-429). In doing so, we might loosely extrapolate the possibility that problems with cognitive flexibility may serve as a prodromal phenotype.***

***Nevertheless, we revised the title, the Conclusions and Relevance section in the Abstract (line 50) to tone down the aging aspect and revised text to state “premature cognitive decline” rather than ‘aging’. Additionally, on p. 16 (line 387), p. 17 (line 410) refers only to a future need for benchmarking cognitive functioning, and p.19 (line 478), and p. 21 (line 509), we refer to the veterans (+) mTBI performance as either having a ‘premature cognitive decline” (line 390) or ‘age-related cognitive decline’ rather than ‘cognitive aging’ per se. We also state that our results occur only in specific domains in addition to recognizing we cannot infer progression of aging. For example, p. 20, line 492-495 states, “another limitation of this study is the cross-sectional design which prevents us from longitudinally tracking the onset or progression of premature cognitive decline nor predicting PD risks in those with mTBI.”***

***We ask the reviewer to consider our comparisons of veterans (+) mTBI to age-matched healthy controls, veterans, decades older Parkinson’s disease subjects in addition to elder normative control data (p.13-14 and Table 1). Our comparison outcomes suggest the presence of some premature cognitive aging ‘effect’ is indeed occurring in our subjects whose mTBI occurred approximately 5.1 +/-1.9 years ago. Thank you.***

Reviewers' comments:

Reviewer's Responses to Questions-

**Comments to the Author**

1. If the authors have adequately addressed your comments raised in a previous round of review and you feel that this manuscript is now acceptable for publication, you may indicate that here to bypass the “Comments to the Author” section, enter your conflict of interest statement in the “Confidential to Editor” section, and submit your "Accept" recommendation.

Reviewer #1: All comments have been addressed

Reviewer #2: (No Response)

2. Is the manuscript technically sound, and do the data support the conclusions?

The manuscript must describe a technically sound piece of scientific research with data that supports the conclusions. Experiments must have been conducted rigorously, with appropriate controls, replication, and sample sizes. The conclusions must be drawn appropriately based on the data presented.

Reviewer #1: Yes

Reviewer #2: Yes

3. Has the statistical analysis been performed appropriately and rigorously?

Reviewer #1: Yes

Reviewer #2: Yes

4. Have the authors made all data underlying the findings in their manuscript fully available?

The [PLOS Data policy](https://nam04.safelinks.protection.outlook.com/?url=http%3A%2F%2Fwww.plosone.org%2Fstatic%2Fpolicies.action%23sharing&data=04%7C01%7Cvicki.nejtek%40unthsc.edu%7C33ba8018805a408f68ae08d96ca7d026%7C70de199207c6480fa318a1afcba03983%7C0%7C0%7C637660290277267300%7CUnknown%7CTWFpbGZsb3d8eyJWIjoiMC4wLjAwMDAiLCJQIjoiV2luMzIiLCJBTiI6Ik1haWwiLCJXVCI6Mn0%3D%7C1000&sdata=uw2ENb0xJ3edfDbQCMTwzV6OYkfZaWil63TgTZjIfnE%3D&reserved=0) requires authors to make all data underlying the findings described in their manuscript fully available without restriction, with rare exception (please refer to the Data Availability Statement in the manuscript PDF file). The data should be provided as part of the manuscript or its supporting information, or deposited to a public repository. For example, in addition to summary statistics, the data points behind means, medians and variance measures should be available. If there are restrictions on publicly sharing data—e.g. participant privacy or use of data from a third party—those must be specified.

Reviewer #1: Yes

Reviewer #2: Yes

5. Is the manuscript presented in an intelligible fashion and written in standard English?

PLOS ONE does not copyedit accepted manuscripts, so the language in submitted articles must be clear, correct, and unambiguous. Any typographical or grammatical errors should be corrected at revision, so please note any specific errors here.

Reviewer #1: Yes

Reviewer #2: Yes

6. Review Comments to the Author

Please use the space provided to explain your answers to the questions above. You may also include additional comments for the author, including concerns about dual publication, research ethics, or publication ethics. (Please upload your review as an attachment if it exceeds 20,000 characters)

Reviewer #1: (No Response)

Reviewer #2: The authors addressed the majority of concerns raised and their revisions have substantially improved their manuscript. There are several remaining issues that need to be addressed:

Abstract
1. Please check this statement for accuracy: “Epidemiologists report 56% of veterans with (+) mild traumatic brain injury (mTBI) develop Parkinson’s disease (PD) within 4.6 to 6.8-years post-injury”.

***Author’s Response***

***We sincerely appreciate and thank the reviewer pointing out the serious omission of RISK we accidentally missed in the first sentence in the Abstract. We surely did not intend to insinuate that there was this overwhelmingly high number of Parkinson’s disease affecting our veterans. We have revised the Importance section in the Abstract lines 30-34. After careful review of our paper, we found in the Introduction, the first sentence correctly stated the 56% increased risk in lines 56-57, and we found no other miscommunications on the ‘risk’ aspect in rest of the paper.***

Introduction
2. Please check this statement for accuracy: “Epidemiologists report that veterans (+) mTBI have a 56% increased risk of developing idiopathic Parkinson’s disease (PD) within ~4.6-years post-injury [1].” I believe that this statement may not be entirely accurate. My understanding of Gardner’s study is that the risk if increased by 56% within (up to) 12 years.

***Author’s Response***

***Gardner et al., 2018 states on page e1774, the initial follow-up for the “entire cohort was 4.6 years and the average time of follow-up was 6.8 +/- 2.24 years”. Then later in the paper, Gardner et al, states on page e1775, that indeed, “that prior TBI is associated with an increased risk of being diagnosed with PD over up to 12 years of follow-up…” Thus, the results were not intuitive, and we believed that the 12-year follow-up referred to any TBI rather than mTBI. However, after additional scanning of the text, we found that the 12-year follow-up actually referred to the 56% increased risk of being diagnosed with PD with mTBI. Therefore, we thank the reviewer for bringing this to our attention and consequently we revised our sentence accordingly (Abstract p 2, line 31, Intro p. 3, line 57).***

3. The new sentence, “In summary, it is uncertain whether global or specific cognitive problems lead to mTBI-related risks for PD onset” is unclear. Are the authors proposing that post-mTBI cognition is the cause for PD onset? Please revise for clarity.

***Author’s Response***

***Thank you for highlighting the fact that this sentence is unclear. We only wanted to state that*** ***the ambiguities in the literature make it difficult to identify a particular characteristic of cognitive functioning that might be used as a prodromal cognitive pre-motor phenotype of mTBI-related risks for PD (p. 4, line 82). We were also concerned that assessing global cognitive functioning (for example) as measured by the Mini Mental State Exam might miss more specific domains of functioning that are problematic for some patients.***

***We deleted this sentence and the next few words in the next sentence with the following replacement p. 4, lines 82-93 … “These ambiguities in the literature make it impossible to identify a particular domain of cognitive functioning that might be used as a prodromal phenotype of mTBI-related risks for PD” and “As such, the gap in our understanding about cognitive problems or premature cognitive decline that may link mTBI to future risks for PD is considerable. Thus, identifying a mTBI-related cognitive characteristic common to both mTBI and PD might help close this gap.”***

***We hope this revision satisfies the reviewer’s concerns.***

Results
4. The normative table was cut-off and was unable to be reviewed.

***Author’s Response***

***Page 16 now reflects the proper format to incorporate a landscape table into a portrait manuscript.***

5. Re: Response to Reviewer 1’s comment 4. There are quite a few studies that have examined cognitive functioning in early-stage PD. For example, Pfeiffer et al., 2014: Acta Neurol Scand 2014: 129: 307–318 DOI: 10.1111/ane.12189.

***Author’s Response***

***We thank the reviewer for bringing this to our attention. In Pfeiffer et al study, it appears that the ‘early-stage’ PD group had mild cognitive impairment which was an exclusion criterion in our study. Moreover, we did not use the CANTAB or most other assessments as used in Pfeiffer et al 2014. So, we believe our outcomes may not correspond with this study.***

***We also did not believe that we could fully use the data to uphold or contrast with our findings as the Pfeiffer study was not a match-control design, and their data could not be used in our younger sample of veterans and healthy controls in terms of similarities with our PD subjects. However, we were able to state on page 20, lines 481-487: “Indeed, in non-demented early-stage PD, Pfeiffer et al., (2014) [55] found 70% of early-stage PD with no previous history of cognitive decline met criteria for a modified mild cognitive impairment diagnosis based on the outcomes from executive functioning/working memory, attention and concentration tests while other cognitive domains were relatively spared. This finding bolsters our previous suggestion that neurocognitive evaluations need to occur early and often for both post- mTBI injury and post-PD diagnosis patients in order to best identify the trajectory affecting the cognitive domains that are most vulnerable to progression of neurodegeneration and possible conversion to cognitive impairment or dementia.***

***Additionally, we had previously cited Marchand et al. 2018 as this study found TMT-B was significantly predictive of future dementia in Parkinson’s disease subjects within 6-years, and we also added DeRoy et al., 2020 who conducted a match-control design with TMT-B and also found TMT-B predicted risks for dementia over a 4.3-year period in non-demented Parkinson’s [p.17-18, lines 419-429].***

***We agree there are other studies as we explain on p. 4 lines 82-90. Nowhere in our manuscript do we suggest that there are no other cognitive studies in early-stage PD. If we have mis-lead the reviewer with our response, that was not our intent and apologize for any confusions.***

***Reviewer 1 suggested we use “…comparative data for other early-stage PD samples on the selection of cognitive tests administered”. In our previous response as shown below, we were***

Unfortunately, we could not find studies examining cognitive testing specifically in early-stage PD… other more advanced stages, yes, but not early-stage. If compared to more advanced stages, then we believe it would add confusion to our study goals and data interpretations.

***strictly speaking in terms of the specific tests and primary outcomes we used were reported mostly in more advanced stages of PD, which, of course would not be an appropriate comparison for our sample. Other pertinent studies are shown in Table 1 along with the normative data to show qualitative comparisons. Overall, we apologize for being unclear about the previous response to Reviewer 1. We did not intend to reflect the actual text in the manuscript, but this statement was more of a talking point - we could not find any text in the manuscript where we stated that there were no other cognitive studies in early-stage PD.***

7. PLOS authors have the option to publish the peer review history of their article ([what does this mean?](https://nam04.safelinks.protection.outlook.com/?url=https%3A%2F%2Fjournals.plos.org%2Fplosone%2Fs%2Feditorial-and-peer-review-process%23loc-peer-review-history&data=04%7C01%7Cvicki.nejtek%40unthsc.edu%7C33ba8018805a408f68ae08d96ca7d026%7C70de199207c6480fa318a1afcba03983%7C0%7C0%7C637660290277267300%7CUnknown%7CTWFpbGZsb3d8eyJWIjoiMC4wLjAwMDAiLCJQIjoiV2luMzIiLCJBTiI6Ik1haWwiLCJXVCI6Mn0%3D%7C1000&sdata=d7L75pZijOS9kTig%2FJ4%2F%2F6sXHKYSS%2Bnum8jVi0b%2Bvvw%3D&reserved=0)). If published, this will include your full peer review and any attached files.

If you choose “no”, your identity will remain anonymous but your review may still be made public.

**Do you want your identity to be public for this peer review?** For information about this choice, including consent withdrawal, please see our [Privacy Policy](https://nam04.safelinks.protection.outlook.com/?url=https%3A%2F%2Fwww.plos.org%2Fprivacy-policy&data=04%7C01%7Cvicki.nejtek%40unthsc.edu%7C33ba8018805a408f68ae08d96ca7d026%7C70de199207c6480fa318a1afcba03983%7C0%7C0%7C637660290277277289%7CUnknown%7CTWFpbGZsb3d8eyJWIjoiMC4wLjAwMDAiLCJQIjoiV2luMzIiLCJBTiI6Ik1haWwiLCJXVCI6Mn0%3D%7C1000&sdata=D%2B66TawA3YQ5A302d3MXGYHhSX5sP0Mpy7gJAOWkDA8%3D&reserved=0).

Reviewer #1: No

Reviewer #2: No

 [NOTE: If reviewer comments were submitted as an attachment file, they will be attached to this email and accessible via the submission site. Please log into your account, locate the manuscript record, and check for the action link "View Attachments". If this link does not appear, there are no attachment files.]

While revising your submission, please upload your figure files to the Preflight Analysis and Conversion Engine (PACE) digital diagnostic tool, [https://pacev2.apexcovantage.com/](https://nam04.safelinks.protection.outlook.com/?url=https%3A%2F%2Fpacev2.apexcovantage.com%2F&data=04%7C01%7Cvicki.nejtek%40unthsc.edu%7C33ba8018805a408f68ae08d96ca7d026%7C70de199207c6480fa318a1afcba03983%7C0%7C0%7C637660290277277289%7CUnknown%7CTWFpbGZsb3d8eyJWIjoiMC4wLjAwMDAiLCJQIjoiV2luMzIiLCJBTiI6Ik1haWwiLCJXVCI6Mn0%3D%7C1000&sdata=lVYwTVjHCWX34k57wZ8ZwVkehuQvQXCNpqk8XKbQV6c%3D&reserved=0). PACE helps ensure that figures meet PLOS requirements. To use PACE, you must first register as a user. Registration is free. Then, login and navigate to the UPLOAD tab, where you will find detailed instructions on how to use the tool. If you encounter any issues or have any questions when using PACE, please email PLOS at [figures@plos.org](mailto:figures@plos.org). Please note that Supporting Information files do not need this step.
